# Gold Nanoparticle Enhanced Proton Therapy: Monte Carlo Modeling of Reactive Species’ Distributions Around a Gold Nanoparticle and the Effects of Nanoparticle Proximity and Clustering

**DOI:** 10.3390/ijms20174280

**Published:** 2019-09-01

**Authors:** Dylan Peukert, Ivan Kempson, Michael Douglass, Eva Bezak

**Affiliations:** 1 Future Industries Institute, University of South Australia, Adelaide, SA 5095, Australia; 2 Division of ITEE, University of South Australia, Adelaide, SA 5095, Australia; 3 Department of Medical Physics, Royal Adelaide Hospital, Adelaide, SA 5000, Australia; 4 Department of Physics, University of Adelaide, Adelaide, SA 5005, Australia; 5 Cancer Research Institute and School of Health Sciences, University of South Australia, Adelaide, SA 5001, Australia

**Keywords:** Monte Carlo, radiosensitization, nanoparticles, proton therapy, radiolysis, clustering

## Abstract

Gold nanoparticles (GNPs) are promising radiosensitizers with the potential to enhance radiotherapy. Experiments have shown GNP enhancement of proton therapy and indicated that chemical damage by reactive species plays a major role. Simulations of the distribution and yield of reactive species from 10 ps to 1 µs produced by a single GNP, two GNPs in proximity and a GNP cluster irradiated with a proton beam were performed using the Geant4 Monte Carlo toolkit. It was found that the reactive species distribution at 1 µs extended a few hundred nm from a GNP and that the largest enhancement occurred over 50 nm from the nanoparticle. Additionally, the yield for two GNPs in proximity and a GNP cluster was reduced by up to 17% and 60% respectively from increased absorption. The extended range of action from the diffusion of the reactive species may enable simulations to model GNP enhanced proton therapy. The high levels of absorption for a large GNP cluster suggest that smaller clusters and diffuse GNP distributions maximize the total radiolysis yield within a cell. However, this must be balanced against the high local yields near a cluster particularly if the cluster is located adjacent to a biological target.

## 1. Introduction

Radiation therapy is a commonly used modality in the treatment of cancer. Radiosensitizers, agents, which increase the biological effect of radiation, which are preferentially delivered to tumor tissue, are being examined as a promising method to improve radiation therapy treatment outcomes [1]. Gold nanoparticles (GNPs) are being considered as potential radiosensitizers due to their high Z (atomic number), and excellent biological compatibility. The high probability for incident radiation to interact within the dense GNP increases the proportion of the radiation’s energy deposited in the local environment of the GNP. Beneficially, GNPs preferentially accumulate in tumor tissue passively via the enhanced permeation and retention effect (EPR) [2,3] causing an increase in the proportion of dose deposited within the tumor tissue. GNPs were thought to only be an effective radiosensitizer for radiation types with interactions with a strong dependence on Z such as photoelectric interactions for kV photons and pair production interactions for photons of megavoltage (MV) energies. However, as discussed below, sensitization has also been observed for protons.

**Figure 1:**
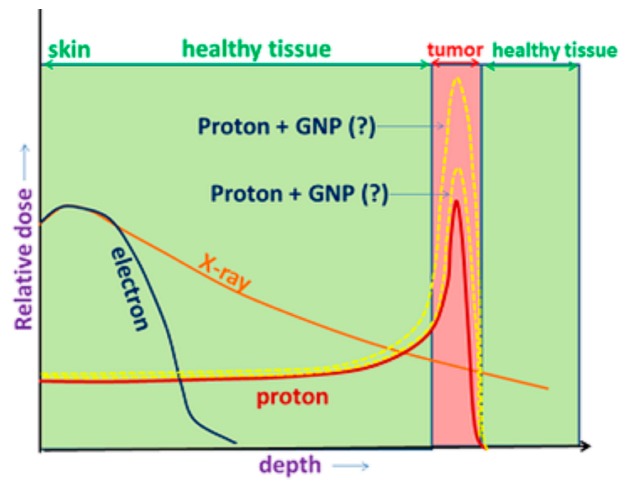
Diagram of the relative dose versus depth in tissue for protons, electrons and MV X-rays shown in red, blue and orange respectively. The proton energy was chosen so that the Bragg peak is collocated with the tumor tissue. The potential for the use of gold nanoparticle (GNP) radiosensitizers to increase the relative dose in the Bragg peak within the tumor tissue is shown.

Proton therapy offers several advantages over conventional MV photon therapy. These advantages originate from the fact that as protons are charged particles, they primarily interact via ionization reactions. For these interactions, the energy deposited within the medium the protons are travelling through increases as the proton loses energy and slows down, producing a peak in energy deposition just prior to the proton stopping, known as the Bragg peak. The primary advantage of proton therapy is the superior dose distribution compared with MV photons resulting from the presence of the Bragg peak. With appropriate treatment planning by placing Bragg peaks throughout the tumor, it is possible to achieve a large reduction in integral dose to the healthy tissue surrounding the treated tumor compared to photon therapy. The relative dose versus depth for protons and MV photons with the proton Bragg peak collocated with the tumor is shown in Figure 1. Another advantage of proton therapy is the increase in the proton’s linear energy transfer (LET) and hence relative biological effectiveness (RBE) as the proton slows down. By planning the treatment to collocate the Bragg peak with the tumor volume, the radiation delivered to the tumor will have a slightly higher RBE (clinically assumed to be 1.1) than the healthy tissue irradiated with protons in the plateau region before the Bragg peak [4,5].

The use of GNP radiosensitizers offer the prospect of an advanced treatment providing improved patient outcomes for photon-based treatments. The ionization interactions of protons however, have a weak dependence on Z and it was not expected that GNPs would be an effective radiosensitizer for proton therapy. This expectation was contradicted by experimental observations, with enhancements being observed in both in-vitro [6,7,8] and in-vivo [9,10] experiments. Experimental observations also provided indications that the enhanced production of reactive species via radiolysis due to the presence of GNPs may play a large role in the observed radiosensitization [8,10,11]. Combining the excellent sparing of healthy tissue of proton therapy with the enhanced biological effect within the tumor from the use of GNP, offers the potential for improved patient outcomes.

Simulations of the biological effect of GNPs have been developed for incident kV and MV photons. Simulations of the macroscopic dose enhancement performed by Cho et al. [12] proved successful in modeling GNP enhancement for kV photons, while cell model simulations [13] considering dose to the nucleus using the local effect model (LEM) [14] to account for non-uniform dose distributions were able to model GNP enhancement for MV photons. Macroscopic dose models [15,16] were unable to model GNP enhancement of proton therapy for GNP concentrations consistent with experiments, while cell models [17] of the dose to the nucleus using the LEM were unable to model GNP enhancement of proton therapy for GNPs distributed within the cytoplasm. Simulations with GNPs within the cytoplasm but not the nucleus are closest to real biological conditions as experiments have shown that GNPs typically can enter the cytoplasm but not the nucleus [18,19]. The reduced radial range [20] of the dose around a GNP for protons compared to photons can explain why no effect is observed for the dose to the nucleus from GNPs in the cytoplasm for proton irradiation.

Indications from experimental observations of the significant role that reactive species play in GNP radiosensitization of proton therapy, combined with the fact that modeling the effect of localized clusters of dose was essential to model GNP radiosensitization of MV photons, suggest that understanding the local distribution of reactive species produced by water radiolysis around a GNP irradiated with protons will aid modeling GNP enhanced proton therapy. As secondary electrons emitted from GNPs irradiated with protons have an even shorter range than for MV photon irradiation, localized clusters of damage to sub-cellular structures will play an important role in the biological effect. Additionally, the diffusion of reactive species allows the potential for an extension to the range of the damage. As such, modeling the radiolysis yield distribution over time around a GNP irradiated with protons would be useful to aid understanding and modeling GNP radiosensitization of proton therapy. Experimental observations have also shown that within a cell GNPs are rarely isolated within the cytoplasm and commonly form clusters [18,19,21]. Accordingly, determining the effect of the proximity of two GNPs on the reactive species’ distribution and the total yields would be a useful aid for modeling GNP radiosensitization of proton therapy.

Several studies have simulated the spherical radial dose enhancement around a GNP irradiated with protons. Walzlein et al. [22] were the first to model the radial dose in water around a single GNP and the equivalent water nanoparticle (WNP). They also found that the radial dose rapidly reduced with distance from the nanoparticle. At shorter radial distances it was found that the dose for the GNP was approximately twice the dose for the WNP. Later simulations by Lin et al. [20], Cho et al. [15] and Tran et al. [23] also modeled the radial dose around a GNP and equivalent WNP. They used the ratio of the radial dose for the GNP and WNP to calculate the radial dose enhancement factor (DEF). It was found that the DEF had a value of 2–3 close to the nanoparticle and reached a plateau of approximately 14 at greater distances from the nanoparticle. Kwon et al. [16] modeled the radial dose in the plane perpendicular to the proton beam. The radial dose was calculated for planes positioned at a range of distances from the center of the nanoparticle along the beam’s path. The difference in dose and the dose enhancement ratio for the GNP compared with an equivalent WNP was calculated. The increase in dose was found to be nearly isotropic around the nanoparticle, however, the inclusion of the dose to the water from the proton beam as well as the emissions from the nanoparticle reduced the maximum DEF to 2.5 and reduced the DEF in regions closer to the proton beam. Tran et al. [23] were the first to model the resulting water radiolysis and reactive species diffusion and interactions around a GNP for proton irradiation. The radial dose in addition to the radial reactive species’ yield were modeled, allowing the radiolysis enhancement factor (REF) to be calculated from the ratio of the yields for the GNP and equivalent WNP. It was found that for up to 10 ns after the start of physical interactions the REF was similar to the DEF, at later times in the simulation the REF was found to be reduced at shorter radial distances. Overviews of experimental observations and simulations of the enhancement of radiotherapy by GNPs have been given by several reviews [24,25,26].

**Figure 2:**
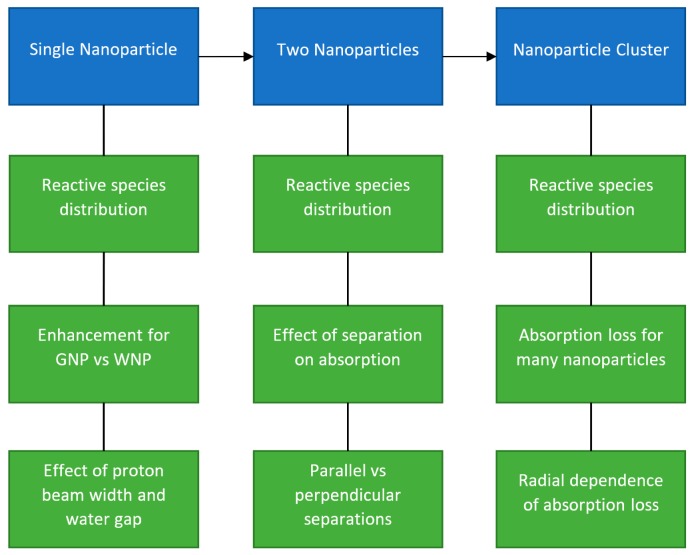
Schematic of the simulation geometries used in this work and the phenomena investigated using the geometries shown in blue and green respectively.

In this work, the spatial distributions of reactive species over time around a GNP and equivalent WNP irradiated with protons were examined using the Geant4 Monte Carlo toolkit [27,28] with the Geant4-DNA very low energy physics and chemistry models [29,30,31,32,33]. The reactive species distributions were scored in 3D bins providing additional spatial information than radial scoring used in previous studies. The spatial distributions of the difference and ratio of the yields were determined along with the effect of the radius of the proton beam and the distance from the proton source to the nanoparticle on the enhancement. The anisotropy of the reactive species distribution around the nanoparticles and how this affects enhancement was evaluated for the first time. The effect of having two nanoparticles in proximity on the combined radiolysis yield’s spatial distribution was examined as well as how the additional absorption of incident protons and secondary electrons from the other nanoparticle affected the total dose and radiolysis yield compared to infinitely separated nanoparticles in the same proton flux. The impact on the absorption from having two nanoparticles in proximity, of whether the nanoparticles were separated in-line with or perpendicular to the beam, was also determined. The reactive species yield distribution and the impact of absorption were also examined for a GNP cluster geometry based on experimental observations. A schematic of the simulations performed is shown in Figure 2.

## 2. Results

### 2.1. Single GNP

**Figure 3:**
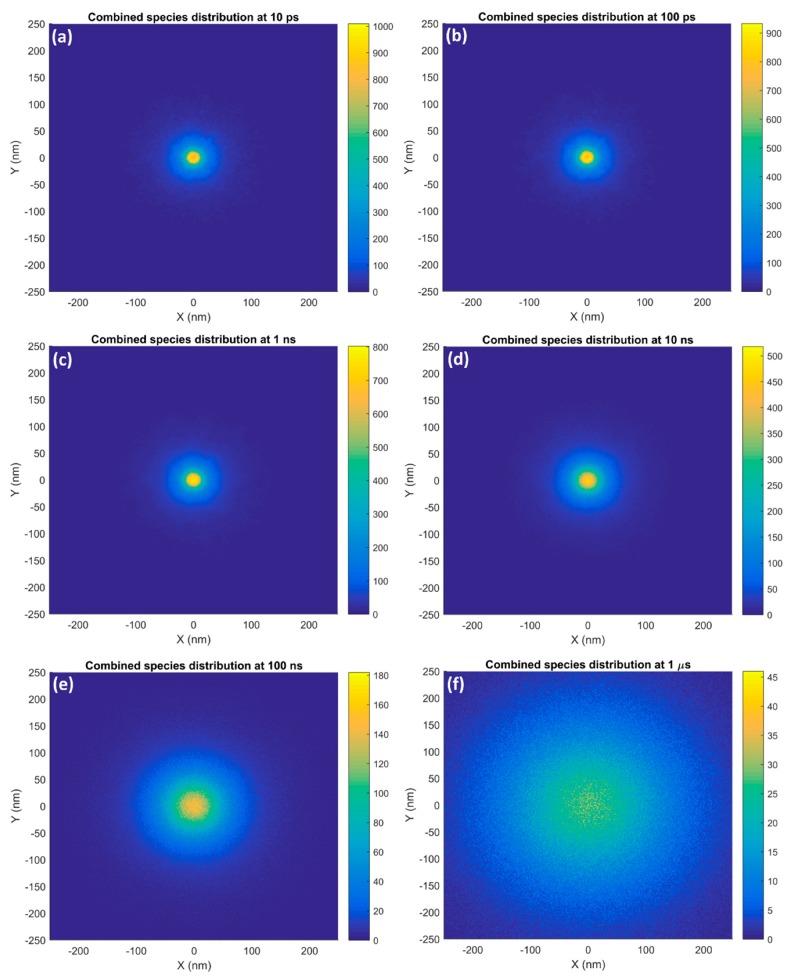
The spatial distribution in the XY plane of the combined reactive species yield around a 15 nm GNP irradiated with a narrow 5 MeV proton beam at (**a**) 10 ps, (**b**) 100 ps, (**c**) 1 ns, (**d**) 10 ns, (**e**) 100 ns and (**f**) 1 µs after the beginning of physical interactions is shown.

The distributions of the combined reactive species in the XY plane around a 15 nm GNP irradiated with a narrow (15 nm) 5 MeV proton beam at 10 ps, 100 ps, 1 ns, 10 ns, 100 ns and 1 µs after the start of physical interactions are shown in Figure 3. At the earlier times in the chemical stage of the simulation, the radiolysis yields were highest at the edge of the nanoparticle’s surface and reduced rapidly with greater radial distance from the nanoparticle. As time progressed the region of the largest yields in the center spread out and became larger and the falloff with distance became less steep. Eventually, at 1 µs the reactive species were spread out in a diffuse cloud. Note that for these simulations, the incident proton beam was generated at the surface of the nanoparticle. As such, the protons did not travel through the water medium before interacting with the nanoparticle.

**Figure 4:**
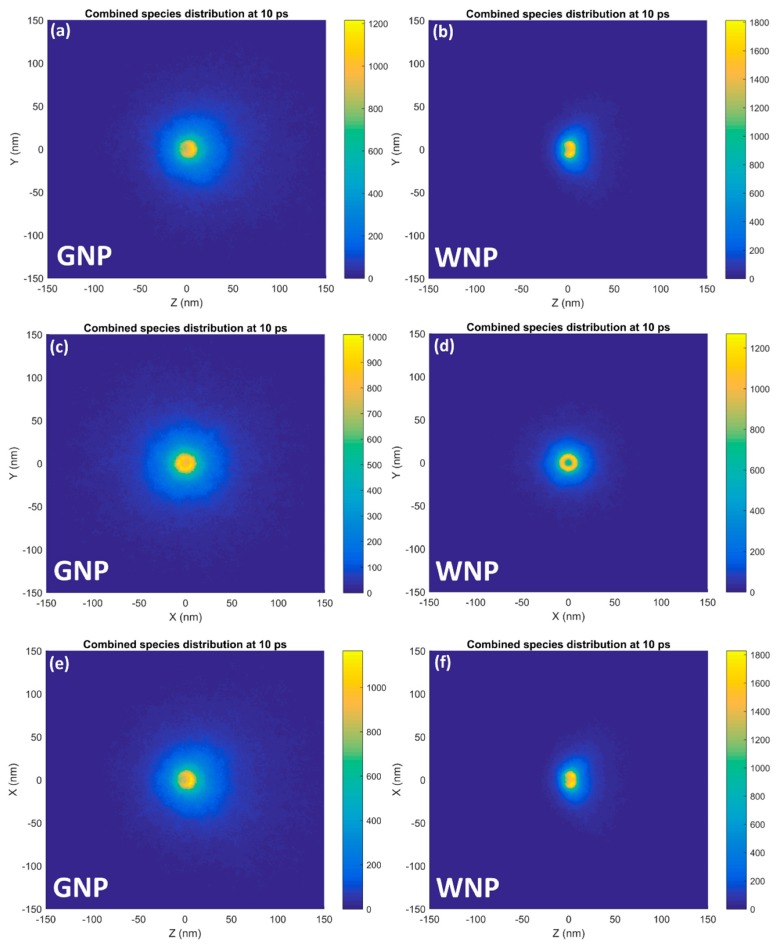
The spatial distribution of the combined reactive species yield around a 15 nm GNP (**a**,**c**,**e**) and equivalent WNP (**b**,**d**,**f**) irradiated with a narrow 5 MeV proton beam in the XZ (**a**,**b**), YZ (**c**,**d**) and XY (**e**,**f**) planes at 10 ps after the beginning of physical interactions is shown.

The distribution of the combined reactive species in the XZ, YZ and XY planes around a 15 nm GNP and equivalent WNP irradiated with a narrow 5 MeV proton beam at 10 ps after the start of physical interactions is shown in Figure 4. In the XY plane, the reactive species were isotropically distributed around the nanoparticle. For both the gold and water nanoparticle in both the XZ and YZ planes, the reactive species distribution was biased in the positive Z direction along the path of the proton beam. The forward bias was greater for the water nanoparticle than the gold nanoparticle.

**Figure 5:**
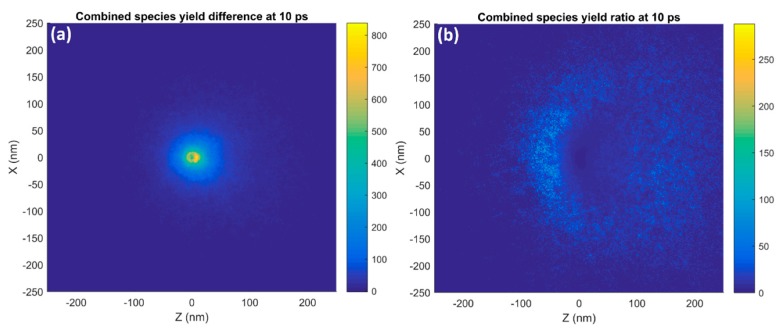
The spatial distribution of (**a**) the absolute difference and (**b**) ratio of the combined species radiolysis yields for a 15 nm GNP compared with an equivalent WNP irradiated with a narrow (15 nm) 5 MeV proton beam in the XZ plane at 10 ps after the beginning of physical interactions is shown.

Figure 5 shows the absolute difference and ratio of the combined reactive species distribution in the XZ plane at 10 ps for a 15 nm GNP and equivalent WNP. The gold nanoparticle resulted in a greater reactive species yield at all points than the equivalent WNP, with the absolute difference in the number of species being greatest at the surface of the nanoparticle and decreasing with greater radial distance from the nanoparticle. The difference distribution had a slight forward bias. While the GNP yield was always greater than the WNP yield, the yield ratio was close to 1 at the nanoparticle’s surface and rapidly increased with greater radial distance reaching a maximum value at a distance of 40–75 nm depending on the direction. The ratio distribution had a strong bias in the negative Z direction.

**Figure 6:**
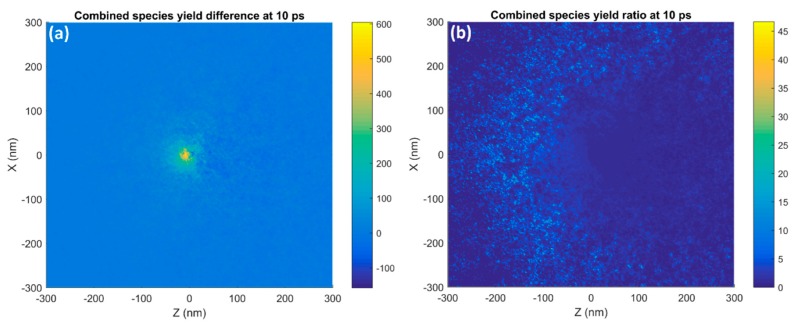
The spatial distribution in the XZ plane at 10 ps after the beginning of physical interactions of (**a**) the absolute difference and (**b**) ratio of the combined species radiolysis yields for a 15 nm GNP compared with an equivalent WNP irradiated with a wide (400 nm) 5 MeV proton beams placed at a greater distance from the nanoparticle to allow for the effect of incident secondary electrons is shown.

Figure 6 shows the absolute difference and ratio of the combined reactive species distribution in the XZ plane at 10 ps for a 15 nm GNP and equivalent WNP irradiated with a wide (400 nm) proton beam at a greater distance from the nanoparticles. These plots show how the difference and ratio changes with an increased beam size and the inclusion of incident secondary electrons compared to Figure 5. A shadowing effect was seen at short distances in the positive Z direction, with a slight reduction in radiolysis yield for the GNP compared to the WNP and a larger region of lower enhancement ratios in the forward direction from the inclusion of incident secondary electrons. Additionally, the enhancement in the backwards direction was increased with the maximum gain being greatest on the negative Z edge on the nanoparticle instead of the positive edge and the range of the enhancement in the backwards direction being increased.

### 2.2. Proximity—Two GNPs

**Figure 7:**
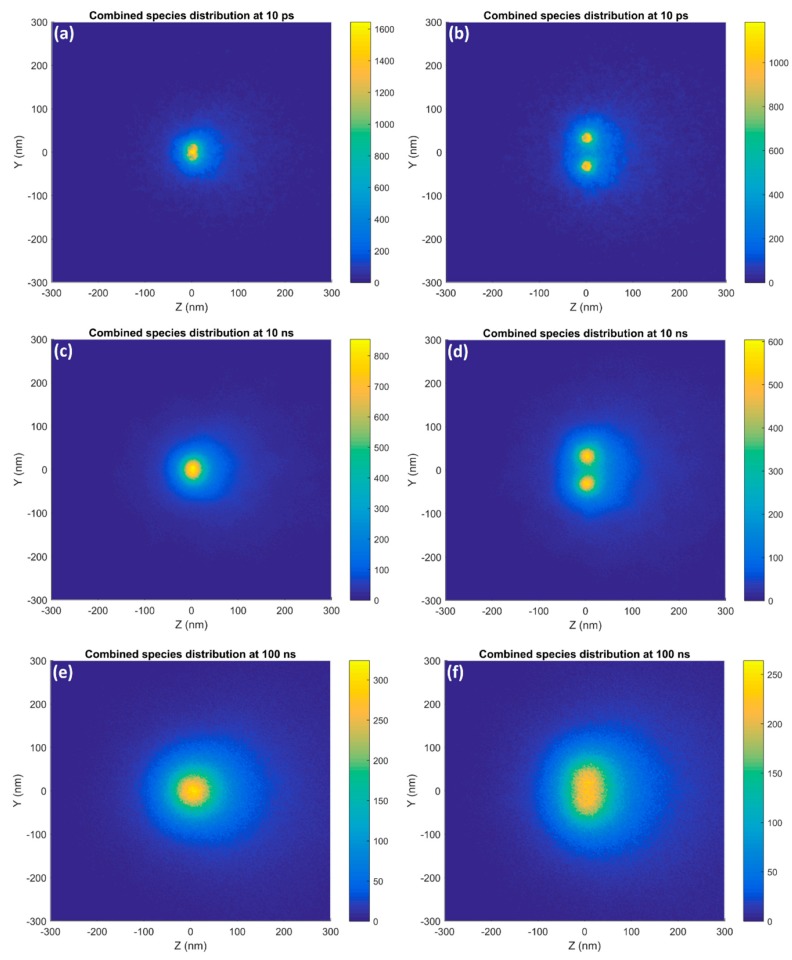
The spatial distributions in the YZ plane of the combined reactive species yield around two 15 nm GNPs separated by 5 nm (**a**,**c**,**e**) and 50 nm (**b**,**d**,**f**) along the Y axis irradiated with a 5 MeV proton beam at 10 ps (**a**,**b**), 10 ns (**c**,**d**) and 100 ns (**e**,**f**) after the beginning of physical interactions is shown.

The distributions of the combined species radiolysis yields at 10 ps, 10 ns and 100 ns in the YZ plane are shown in Figure 7 for two GNPs separated by 5 and 50 nm along the Y axis perpendicular to the beam direction. For the GNPs separated by 5 nm at 10 ps after physical interactions the distributions were similar to two single nanoparticle distributions with a slight overlap resulting in a hot spot of high radiolysis yield between the nanoparticles. By 10 ns the diffusion results in the nanoparticle’s distributions merging with an oblong shape in the Y axis and by 100 ns the distribution became similar to that for a single nanoparticle. The larger 50 nm separation results in reactive species distributions similar to two single nanoparticle distributions overlapped with the only combined effect being an extension of the lower yield regions between the nanoparticles for 10 ps and 10 ns after physical interactions. By 100 ns the distributions had merged into one, which was oblong in the Y axis.

**Figure 8:**
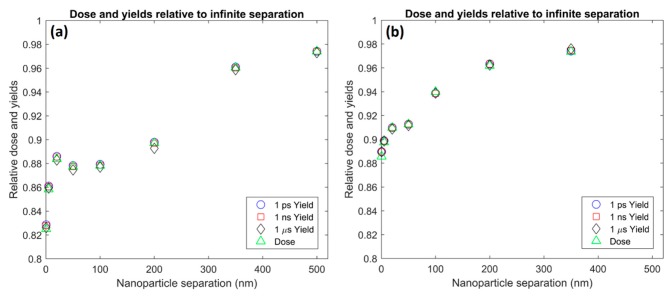
The dependence on the separation distance of dose (triangles) and radiolysis yield at 1 ps (circles), 1 ns (squares) and 1 µs (diamonds) for two nanoparticles separated (**a**) along the beam direction and (**b**) perpendicular to the beam direction relative to the dose and yields for infinitely separated nanoparticles.

The effect of the separation distance, for nanoparticles separated along the proton beam direction, on the reduction in dose to the surrounding water and the resulting combined radiolysis yield at 1 ps, 1 ns and 1 µs after the beginning of physical interactions is shown in Figure 8a. For a small separation of 0.5 nm, a relative dose and radiolysis yield of less than 83% was observed. This indicates a large loss of over 17% of the dose and radiolysis yield primarily due to additional absorption of secondary electrons by the second nanoparticle. At first, as the separation increased to 20 nm, the absorption rapidly reduced to 11%. For separation distances of between 20 and 100 nm a plateau in absorption at a value of 11–12% was observed. Finally, as the separation distance increased further, the absorption decreased with a diminishing trend towards zero, but still on the order of ~2% at a separation of 500 nm.

The effect of the separation distance, for nanoparticles separated in a direction perpendicular to the proton beam, on the reduction in dose to the surrounding water and the resulting combined radiolysis yield at 1 ps, 1 ns and 1 µs after the beginning of physical interactions is shown in Figure 8b. The dependence of absorption on separation was similar to the separations along the beamline, however, there were some differences. The maximum absorption at a separation of 0.5 nm was less than 12%, this was considerably lower than for separations parallel to the beam. Additionally, the initial loss of absorption up to a separation of 20 nm was slower than for the parallel separation. For perpendicular separations, the plateau was shorter only lasting to 50 nm separations and occurred at a lower absorption value of 9%. Finally, as the separation distance increased further, the absorption decreased towards zero. This decrease started earlier for the perpendicular separation compared to the parallel separation.

### 2.3. GNP Cluster

**Figure 9:**
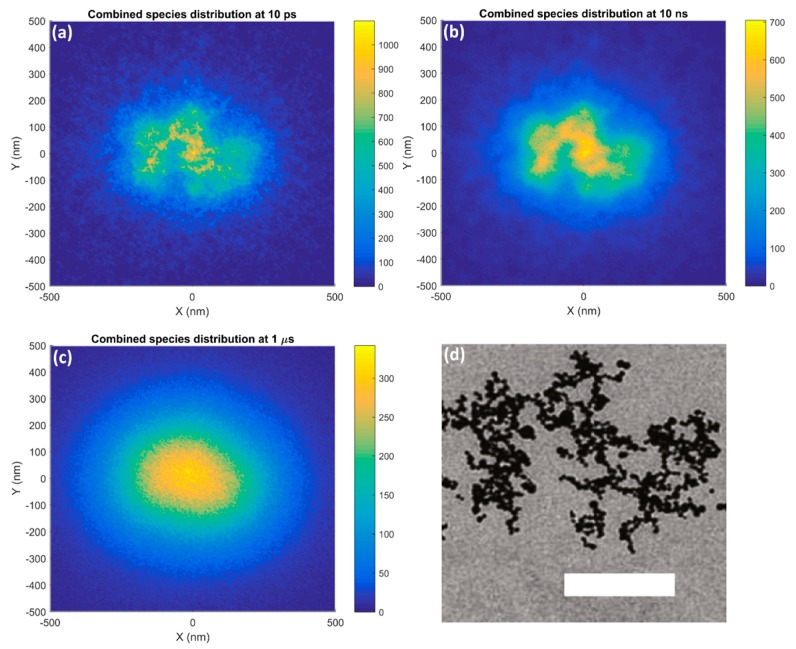
The spatial distributions in the XY plane of the combined reactive species yield around a 500 nm GNP cluster irradiated with a 5 MeV proton beam at (**a**) 10 ps, (**b**) 10 ns and (**c**) 1 µs after the beginning of physical interactions and (**d**) the TEM image (adapted from Chen et al. [21]) used to build the cluster are shown. The bar in the TEM image has a length of 200 nm.

The distributions of reactive species in the XY plane at 10 ps, 10 ns and 1 µs for a 500 nm GNP cluster consisting of 1,402 GNPs each of 15 nm diameter are shown in Figure 9 alongside the TEM image from Chen et al. [21] used to build the cluster. The reactive species distribution at 10 ps corresponds well with the distribution of GNPs within the cluster built from the shown TEM image. The regions of higher yield typically corresponded with the locations of the GNPs, however the individual GNPs were not resolvable. At 10 ns only regions of higher and lower GNP concentrations within the cluster could be distinguished while by 1 µs the distribution approached that for a single large nanoparticle with the cluster substructure no longer visible.

**Figure 10:**
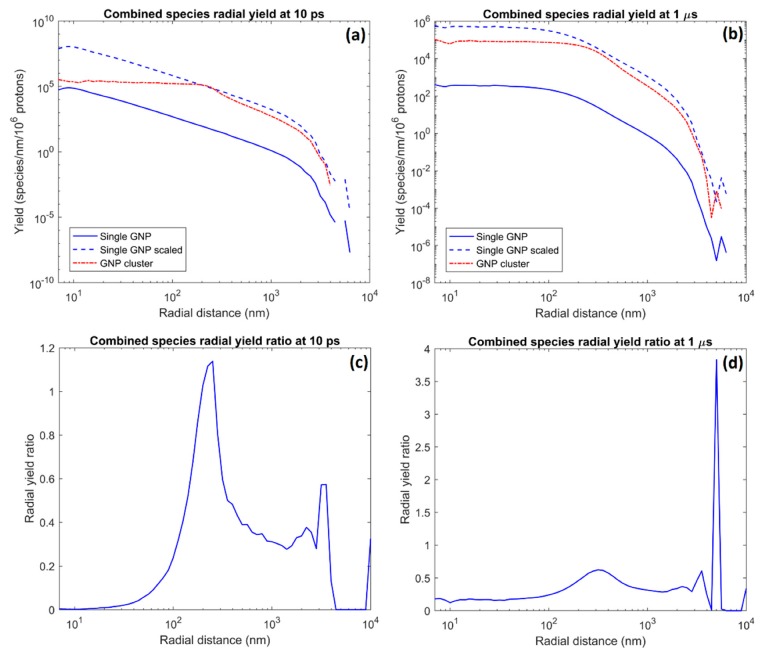
The radial combined reactive species yields for a GNP cluster and single nanoparticle (**a**,**b**) and the ratio of the radial yield for the cluster with the scaled single GNP yield (**c**,**d**) at 10 ps (**a**,**c**) and 1 µs (**b**,**d**) after the beginning of physical interactions.

The radial yield per incident proton flux per water volume for a GNP cluster, single GNP and single GNP scaled by the number of GNPs within the cluster (i.e., 1,402 times the single GNP yield) along with the radial ratio of the GNP cluster yield with the scaled single GNP yield are shown in Figure 10. The radial yield for the GNP cluster was always greater than that for a single GNP, however the cluster yield was lower than the scaled single GNP yield at most radial distances and particularly within the cluster. For the single nanoparticle at 10 ps the radial yield decreased consistently from the nanoparticles radius to 2 µm at which point the rate of yield falloff increased significantly. For the GNP cluster the radial yield was approximately constant within the nanoparticle and had similar trends to the single nanoparticle at greater radial distances. At 1 µs the single GNP yield was approximately constant to radial distances of 100 nm. The single GNP radial yield at greater radial distances and the cluster yield at 1 µs had similar behavior to the yield distributions at 10 ps.

Comparison of the total radiolysis yield and dose for a GNP cluster with a single GNP scaled for number of nanoparticles and incident proton flux showed that the yields at all times and the dose was reduced by 60% for the cluster compared to the single nanoparticle primarily due to the increased absorption of secondary electrons. The radial ratio plots show that the lowest ratios and hence greatest absorption occurred within the radius of the GNP cluster. The yield ratio decrease within the radius of the cluster was greatest at 10 ps and was reduced at 1 µs. For the cluster yield at 10 ps there was an increase in the yield ratio just beyond the cluster radius, which resulted in the ratio being above 1, indicating a brief yield enhancement for the cluster compared to the scaled single GNP. For the yield at 1 µs the increase was reduced and the ratio remains below 1. After the first peak at the cluster radius the yield ratio was between 0.3 and 0.4 until a second peak of 0.6 at 3.5 µm and 3.8 at 5 µm at 10 ps and 1 µs respectively. Note that while a high ratio was reached at the second peak at 1 µs the yield at the large distance from the origin was very low.

## 3. Discussion

### 3.1. Single GNP

At earlier times in the simulation the distribution of the reactive species around a single GNP, as shown in Figure 3, closely follows the dose distribution from the interactions of the secondary electrons. As the electron energy spectrum predominantly consisted of very low energy electrons of less than 200 eV with limited range, the peak in dose and hence reactive species yield occurred close to the nanoparticle’s surface. The reduction in yield at 10 ps with greater distance from the nanoparticle was largely due to a reduction in dose at greater distances from the nanoparticle as fewer of the emitted electrons had sufficient range to contribute. At later times in the chemistry simulation the diffusion of the species began to play a significant role on the distribution. As the species diffused, the region of highest yield spread out. At the same time, the maximum yield at a given point decreased due to the diffusion and the species being consumed in chemical reactions, which were particularly prevalent in the regions of highest species concentration.

For the reactive species distributions of both the GNP and equivalent WNP shown in Figure 4, it was observed that the species distribution was isotropic perpendicular to the beam direction with a forward bias in the direction of the incident proton beam. The forward bias was observed to be more prevalent for the WNP than the GNP. By Fermi-Eyges scattering theory, the level of scattering was dependent on the atomic number of the scattering material. As gold has a high atomic number it is an effective scattering material, gold foil is used in many applications where the scattering of a particle beam is desired. While the electrons produced within the gold and water nanoparticles likely had similar initial forward biases the increased scattering power of the GNP results in a diminished forward bias by the time the electrons left the nanoparticle due to the scattering of the electrons within the gold spreading out the angular distribution.

The reactive species concentration around the GNP was higher than that for the WNP at all points. The absolute difference distribution shown in Figure 5a had a slower fall-off with distance from the nanoparticle and was less forward biased than the GNP distribution. This was due to the fact that the higher energy secondary electrons for the GNP result in a slower falloff in species concentration with distance than for the WNP. As such, the difference decreased more slowly than the GNP yield. Additionally, as the WNP distribution was more forward biased than the GNP distribution, the difference in the forward direction was reduced, resulting in only a slight forward bias. While the largest absolute difference in yield occurred near the nanoparticle’s surface, the ratio of the yield increase at this point was fairly small as shown in Figure 5b. Due to the faster falloff for the WNP than the GNP the yield ratio increased with greater distance from the GNP until the yield for the GNP began to fall off to a large degree as well. The reduced forward bias for the GNP compared to the WNP caused the yield increase ratio to be reduced in the positive Z direction and increased in the negative Z direction.

The incorporation of a wider incident proton beam and increasing the distance the protons had to pass through water to reach the nanoparticle resulted in an incident particle beam closer to that expected in a treatment scenario with the inclusion of incident secondary electrons. This caused a large change in the difference and ratio of the radiolysis yield around a GNP compared to the equivalent WNP as shown in Figure 6. The low energy incident electrons were likely to be absorbed or significantly scattered by interactions within the GNP while the same electrons were more likely to pass through the equivalent WNP without a large amount of absorption or scattering. This produced a shadowing effect where there was actually a region forward of the nanoparticle along the beam direction where the radiolysis yield was equal to or slightly lower than the WNP. The enhancement ratio was higher in other directions due to additional electrons scattered at large angles from the GNP. Eventually an enhancement was observed in the forward direction at greater distances from the nanoparticle for the GNP due to the higher energy secondary electrons produced by proton interactions within the gold having a greater range. The increased energy loss of the incident protons within the GNP is partially counterbalanced by the increased absorption of incident secondary electrons released by previous interactions within water proximal to the nanoparticle. This results in simulations considering only proton interactions within a GNP slightly overestimating the total enhancement and a large overestimation of the enhancement just forward of the nanoparticle. Simulations considering only proton interactions in the nanoparticle provide pure results useful for understanding fundamental dependencies however the irradiation conditions play an important role and for uses where biological outcomes are modeled it is essential that the irradiation conditions are similar to those for treatment.

### 3.2. Proximity—Two GNPs

The combined reactive species distributions of two nanoparticles shown in Figure 7 depend strongly on the proximity and the time after the start of physical interactions. Nanoparticles in close proximity resulted in ‘hotspots’ in the dose and initial radiolysis yields between the nanoparticles. However, at greater separations no hotspot between the nanoparticles was observed. This was due to the rapid falloff in dose and radiolysis yield with distance from the nanoparticle. As such, the effect observed in this case was that the overlapping distributions could extend the low yield distribution envelope between the nanoparticles. As the simulation time advanced, the increasing diffusion of the reactive species caused the distributions to spread out. At later times, the distributions around the two nanoparticles would merge to form a single oblong-shaped distribution with the longer axis along the separation of the nanoparticles. With even greater time, the distribution became effectively isotropic eliminating any indication that it was produced by two nanoparticles. The time for the distributions to merge and eventually form an isotropic distribution increases with the distance the nanoparticles were separated by as the species from the separate nanoparticles required more time to diffuse together. While eventually the reactive species distribution for two nanoparticles separated by smaller distances would resemble the distribution of a single larger nanoparticle, modeling separate nanoparticles was advantageous as features such as hotspots between nanoparticles, the oblong shape of the distribution at intermediate times and the yield envelope extension would be lost with a single nanoparticle approximation.

The dependence of absorption losses for both dose and radiolysis yields due to two nanoparticles in proximity on the separation distance of the nanoparticles has complex behavior. It is observed in Figure 8 that the absorption first fell with separation to a lower value, then reached a plateau before falling again, approaching zero. This behavior was due to the combination of three factors; geometric effects, scattering effects and secondary electron range effects.

At separation distances of less than 20 nm, geometric effects are dominant. The rapid decrease in absorption is caused by the solid angle representing the other nanoparticle decreasing with separation. At these shorter separation distances scattering plays a limited role as electrons are not likely to be scattered by a large angle while traversing between the nanoparticles. Additionally, electrons emitted by one nanoparticle, which are scattered towards the other nanoparticle are more likely to be absorbed by the nanoparticle they were emitted from due to the short distance between the nanoparticles, resulting in a shielding effect. Due to the short separation distance, range effects are also limited as almost all emitted secondary electrons have sufficient range to reach the other nanoparticle.

A plateau was observed from 20 to 50 nm and 20 to 100 nm for perpendicular and parallel separations respectively. The plateau was due to the increasing prevalence of scattering effects counter balancing the geometric effect. As the separation distance increased, the average scattering angle experienced by electrons reaching the separation distance from the nanoparticle increased. While electrons that were travelling towards the other nanoparticle were scattered away from the other nanoparticle, electrons that were travelling in a direction that would have missed the other nanoparticle could be scattered towards the other nanoparticle. The net effect of scattering was an increase in absorption as the proportion of electrons emitted travelling towards the other nanoparticles was small for larger separations so the effect from electrons being scattered towards the other nanoparticle was dominant. The increase in absorption from scattering effects balanced the decrease in absorption from geometric effects causing an absorption plateau.

As the separation increased the proportion of emitted secondary electrons that have sufficient range to reach the other nanoparticle and be absorbed decreased. This range effect became dominant at greater distances and caused a reduction in absorption beyond the plateau. Eventually, the separation became greater than the maximum range of almost all emitted electrons and absorption approached zero. For parallel separations the absorption and scattering of the proton beam by the first nanoparticle reduced the proton flux incident on the second nanoparticle further along the beam’s path resulting in an additional reduction in the dose and radiolysis yield compared with infinitely separated nanoparticles.

While the overall dependence of the absorption on separation distance for two nanoparticles in proximity was similar for separations perpendicular and parallel to the beam, some differences were observed. For all separations modeled a lower absorption of both dose and radiolysis yield was observed for the perpendicular separation compared to the parallel separation. This was due to the forward bias of the emitted secondary electrons. For the parallel separation the secondary electrons from the nanoparticle closest to the beam source emitted in the forward direction travel towards the other nanoparticle resulting in greater absorption than for perpendicular separations where the forward directed electrons were emitted away from the other nanoparticle. Additionally, the absorption plateau ended at a lower separation distance for perpendicular separations than parallel separations. This was caused by the loss of energy experienced by electrons scattered within the gold nanoparticle causing lower average energies and hence ranges for secondary electrons emitted perpendicular to the beam than those emitted along the beam direction. This results in range effects being stronger and becoming predominant at lower separations resulting in the earlier end of the plateau due to reduced absorption for perpendicular separations.

### 3.3. GNP Cluster

In the combined reactive species distribution for the GNP cluster, shown in Figure 9, at 10 ps the regions of higher reactive species yield closely corresponded with the cluster structure from the TEM image. The individual GNPs were not resolvable due to the overlap of many radial reactive species distributions from each nanoparticle. As the chemistry stage progressed the diffusion of the reactive species obscured the cluster structure. By 10 ns only regions of high and low reactive species yield corresponding to areas of high and low GNP density were seen and by 1 µs the distribution was similar to that for a large nanoparticle with no cluster structure visible.

Dose and radiolysis yields for the GNP cluster were only 40% of the single GNP yield for the same incident proton flux scaled to the number of GNPs within the cluster. This shows that the GNP cluster loss 60% of the potential dose and reactive species yield in water to increased absorption by the nanoparticles within the cluster. The absorption loss could be minimized by increasing the separation between nanoparticles within a cell. This may be achieved by designing nanoparticles to form smaller clusters within the target cells or to remain diffuse within a cell to maximize the total dose to water and resulting water radiolysis. Molecular simulations have investigated the effect of nanoparticle functionalization on aggregation [34] and have the potential to guide nanoparticle design to reduce aggregation to limit absorption losses.

For the radial absorption, shown in Figure 10, at 10 ps it was observed that absorption approaches 100% within the center of the GNP cluster. The yield ratio increased rapidly as the radial distance approached the cluster radius and there was a slight enhancement just beyond the GNP cluster radius. This behavior was due to the yield being effectively constant within the cluster (although at a lower yield cause by increased absorption) due to the overlapping of many single nanoparticle distributions while falling off rapidly for the single nanoparticle. The reduced radiolysis yield within the cluster would result in only a limited biological effect due to the absence of biological targets within the GNP cluster. Additionally, as nanoparticle clusters are often contained within vesicles within a cell, the enhancement just beyond the cluster radius had the potential to damage the vesicle membranes. Rupture of the membrane will release the reactive species and enable additional biological damage to the cell. The yield ratio fell again beyond the cluster before a smaller peak at the maximum range of the emitted secondary electrons. This second peak was due to GNPs at greater radial distances from the center of the cluster, which caused the radial range falloff to begin at a slightly longer range than for the single nanoparticle at the origin.

The radial yield at 1 µs was similar to that at 10 ps. The diffusion of the reactive species caused more species to enter the cluster while also flattening the single GNP radial yield to become more like that for the cluster. These two effects caused the yield ratio to be increased within the cluster radius, however this remained the region of greatest absorption. The diffusion of the species reduced the peak in the ratio just beyond the nanoparticle radius and an enhancement was no longer seen. The increased range from diffusion increased the second peak due to amplifying maximum range effects. However it must be noted that while a high yield enhancement ratio was seen, the absolute yield was very low so the absolute gain in reactive species yield was small.

The radial yields show that the yield from the GNP cluster was always considerably greater than the single GNP yield. The increased local gold mass due to a cluster maximizes the dose and radiolysis yield within the local volume surrounding the cluster. While the enhancement of dose to water and the resulting radiolysis is less efficient for a cluster than diffuse GNPs due to increased absorption, if the cluster can be delivered to a critical biological target within a cell, a GNP cluster will result in greater local damage to the target.

## 4. Materials and Methods

**Figure 11:**
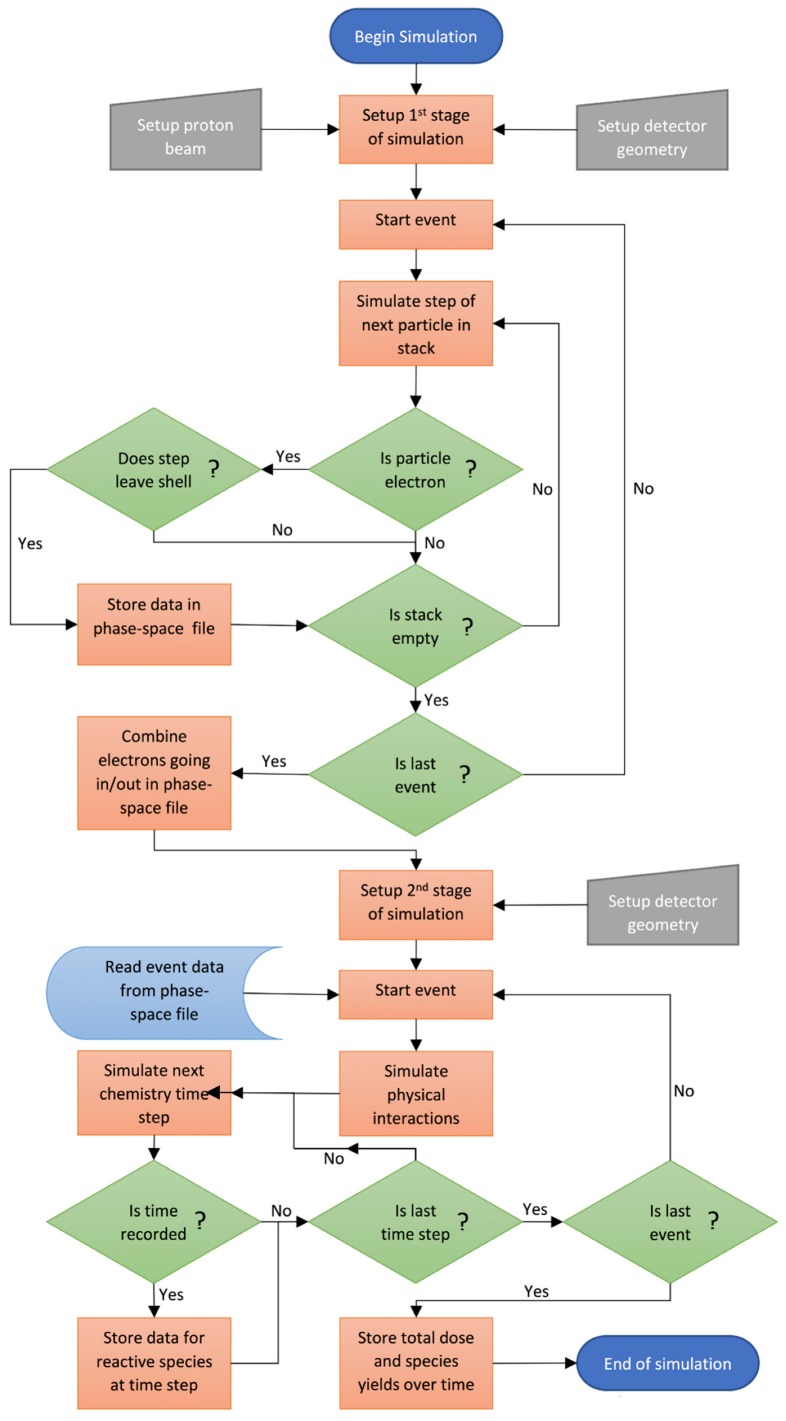
Flowchart of the simulation process.

Simulations were performed using the Geant4 open source Monte Carlo toolkit [27,28]. Simulations were performed in two stages. In the first stage, the physical interactions of the incident protons within the nanoparticle(s) and surrounding water volume were modeled using the Livermore low energy physics models. Secondary electrons leaving or entering any of the nanoparticles were scored in a phase space file. The second stage of the simulation used the phase space file as an input to model the physical interactions of the secondary electrons in water and the resulting water radiolysis including diffusion and interactions of reactive species using the Geant4-DNA physics and chemistry models [29,30,31,32,33]. The dose, radiolysis yield and spatial distribution of the reactive species at several time points throughout the chemistry stage were recorded. A flowchart of the simulation process is shown in Figure 11. The simulations were performed with a computer equipped with a 3.4 GHz AMD Ryzen 1950X CPU with 16 cores and 32 computation threads and 32 GB of RAM. Utilizing the multi-threaded capability of Geant4, the first stage of the simulation took 20–120 min of computation time, while the second stage took 16–24 h.

**Figure 12:**
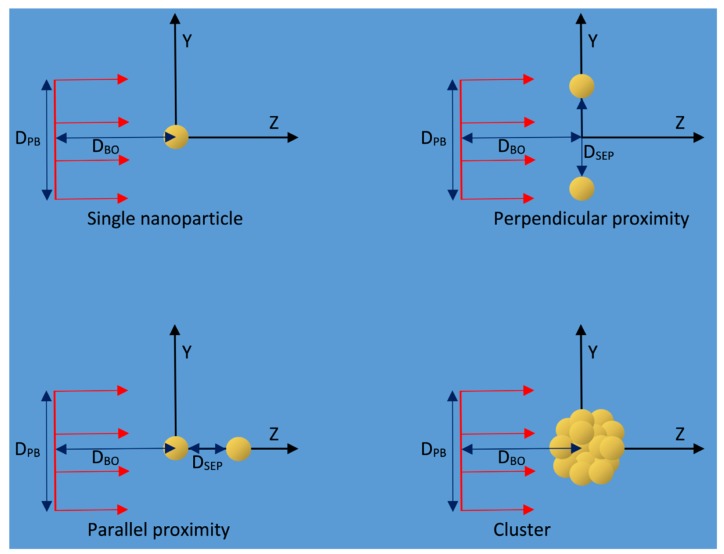
Diagram of the simulation geometry used in the first stage of the simulation. The positions of the nanoparticles relative to the incident proton beam (red arrows) for the different cases simulated are shown. D_PB_, D_BO_ and D_SEP_ are the proton beam diameter, the distance from the proton beam source to the origin and the separation between the nanoparticles respectively. The phase space scoring shells around each nanoparticle are not shown. Diagram is not to scale.

In the first stage of the simulation the geometry consisted of a 2 µm × 2 µm × 2 µm water world volume within which spherical, uncoated gold nanoparticle(s) with a diameter of 15 nm were placed. Simulations were also performed with equivalent water nanoparticles in place of the GNPs for enhancement calculations. Each nanoparticle was surrounded by a 0.1 nm thick phase space scoring shell consisting of water. The placement of the nanoparticles and the incident proton beam for the first stage of the simulations performed is shown in Figure 12 and the proton beams simulated are detailed in Table 1.

A sensitive detector setup within the phase space scoring shells recorded the position, momentum direction, energy, particle track ID, parent track ID and event ID of all electrons that left the scoring shell to enter either the world volume (encompassing the nanoparticle and scoring shell volume(s)) or the enclosed nanoparticle volume. An additional variable tracked which volume the electron entered upon leaving the scoring shell and hence whether the electron was leaving or entering the nanoparticle’s surface. The data for all electrons leaving or entering the nanoparticle were stored in a phase space file. Only electrons were stored as they comprise over 99.9% of secondary particles leaving the GNP.

Electrons entering the nanoparticles were scored to account for the absorption of the secondary electrons by the nanoparticles and the resulting reduction in the dose to water and radiolysis. The electrons leaving a nanoparticle for an event were compared with the electrons entering a nanoparticle. If an incoming electron had a track ID or parent track ID that matched an outgoing electron and had energy less than or equal to the outgoing electron, the energy deposited within water was determined by subtracting the energy of the incoming electron from the energy of the outgoing electron. A new phase space file consisting of only the outgoing electrons was produced, with a new data point giving the energy at which they were absorbed within a GNP. For electrons that were not absorbed this value was set to zero.

In the second stage of the simulation the physical interactions of the secondary electrons within a 2 mm × 2 mm × 2 mm water volume and the resulting water radiolysis, reactive species diffusion and chemical reactions were modeled using the Geant4-DNA physics and chemistry models. The phase space file of outgoing electrons was used as an input to generate the electrons within the second stage of the simulation. The physical interactions of the electrons were modeled until either the electron reached the energy at which it was absorbed in the first stage of the simulation or all of the electron’s energy was deposited in the medium. This preserves both the total dose deposited in water and the electron energy at which the deposition occurred which is essential as the rapidly increasing LET as an electron loses energy and stops results in large changes to the radiolysis yields.

The second stage of the simulation records the total dose and the yield of the seven modeled reactive species (OH, OH^−^, H_2_, H_3_O^+^, H, H_2_O_2_ and e^−^_aq_) throughout the chemistry stage of the simulation. Additionally, the position of each reactive species and its type were recorded at six time points within the chemistry stage of the simulation; 10 ps, 100 ps, 1 ns, 10 ns, 100 ns and 1µs after the start of physical interactions. To display the reactive species positional data, the positions of the reactive species were scored in a 3D mesh with 500 by 500 by 500 bins with a length of 1 nm for narrow beam single nanoparticle simulations, 300 by 300 by 300 bins with a 2 nm length for wide beam single nanoparticle and nanoparticle proximity simulations and 200 by 200 by 200 bins with a 5 nm length for cluster simulations. To display the distribution in a 2D plot the bins along the X, Y or Z axes were integrated to produce three 2D projected distributions in the YZ, XZ and XY planes for each 3D reactive species distribution. The radial yield distribution was also determined by binning the species in spherical shells around the origin with the radius of the shell being increased on a logarithmic scale from 1 nm to 10 µm with 10 bins per decade. The spatial distribution of enhancement for reactive species yield was found by normalizing the measured species distributions for the GNPs and WNPs to the same incident proton flux and taking the difference and ratio for each bin of the histogram.

### 4.1. Single GNP

Spatial and temporal distributions of the reactive species yield over time for a narrow incident proton beam incident upon a 15 nm GNP and an equivalent water nanoparticle of the same size were simulated. The distributions were compared to determine the spatial distribution of the enhancement in reactive species yield by a GNP. The effect of proton beam size and incident secondary electrons was determined by repeating the single nanoparticle simulations with a larger proton beam size with increased water separation between the beam source and the nanoparticle. A 15 nm GNP was chosen for these and following simulations as this size was found to be an ideal balance between the gain in enhancement due to greater gold mass and the loss in enhancement due to self-absorption for larger nanoparticles. A proton energy of 5 MeV was used in these and following simulations for consistency with the lower energy protons within the Bragg peak, which in treatment plans is collocated with the tumor. Additionally below 5 MeV we observed a large reduction in enhancement due to increased self-absorption.

### 4.2. Proximity—Two GNPs

To determine how the proximity of two nanoparticles affect the combined reactive species distributions simulations were performed with two 15 nm diameter nanoparticles in a water volume with separation distances (D_SEP_) of 0.5, 5, 20, 50, 100, 200 and 350 nm for both separations perpendicular to and in-line with the beam as well as an additional simulation for a 500 nm separation in-line with the beam. The effect on the total yield due to the additional absorption of secondary electrons by the other nanoparticle was determined by calculating the ratio of the yield for the two nanoparticle simulations to twice the yield for one nanoparticle for the same incident flux, representing an effectively infinite separation. The relative reduction in yield for the nanoparticles in proximity compared to infinite separation gives the loss due to additional absorption by the other nanoparticle. The simulations were performed with the nanoparticle separation both along the path of the proton beam and perpendicular to the beam direction to determine how any anisotropy in the emitted secondary electrons would alter the effect of nanoparticle proximity. For perpendicular separations of 200 and 350 nm, a wider beam was used to avoid penumbra effects. To account for any difference in incident flux at the different nanoparticle positions, single nanoparticle simulations were performed at the positions for 0 and 100 nm perpendicular separations and the interpolated single nanoparticle results were used to calculate the absorption. For the wide beam perpendicular separations, the single nanoparticle results for the simulated separations were calculated with the wider incident proton beam and used to calculate the absorption. For separations in-line with the beam single nanoparticle simulations were performed at positions of 0 and 500 nm along the Z axis and the yield used to calculate the absorption was found by giving equal weight to the yield at 0 and the interpolated yield for a given separation. The formulas used to calculate the weighted single nanoparticle dose and yield for relative dose and yield calculations are shown in Table 2.

### 4.3. GNP Cluster

Simulations of a nanoparticle cluster were performed to determine the reactive species distribution and the effects of absorption on the yields for a nanoparticle geometry corresponding with experimental observations of GNP distributions within cells. A cluster with a 500 nm diameter containing 1402 nanoparticles each of 15 nm diameter was constructed from a transmission electron microscopic (TEM) image of GNP distributions in a EMT-6 cell [21] shown in Figure 9d. The nanoparticle distribution within a slice of the cluster was extracted from the image. For regions where individual nanoparticles were not resolvable the number of nanoparticles within the region was estimated and their positions were distributed throughout the region. As the TEM image was of a 100 nm thick slice the depth of the nanoparticles within the slice was set to be a random distribution. Overlap checks were performed and the depth within the slice for overlapping nanoparticles was reassigned until all overlaps were removed. The cluster was built by stacking five cluster slices vertically and removing all nanoparticles outside of a 250 nm radius from the center of the middle slice. For comparison simulations of a single 15 nm GNP at the origin were run with the same proton beam configuration as the cluster simulations. The absorption due to the cluster was found by scaling the yields to the same incident flux and comparing the cluster yield with 1,402 times the single nanoparticle yield representing the yield for the GNPs of the cluster at effectively infinite separation. To measure the potential range extension effects from the overlapping yields of many nanoparticles, the yield comparison was also performed for the radial distributions.

The number of incident protons simulated was chosen to balance statistical uncertainty with reasonable computation time requirements. The number of protons modeled was determined by the probability of a proton interacting with a GNP, with larger proton beams requiring more incident protons. For comparisons, the simulations were setup so that the number of secondary electrons leaving the nanoparticles was similar to ensure similar statistical uncertainties for the compared quantities.

## 5. Conclusions

The simulations performed in this work modeled the spatial distribution of reactive species around GNPs over time with a full 3D analysis of the yield distributions and enhancement performed for the first time. It was found that at 1 µs after physical interactions the reactive species diffused up to a few hundred nanometers away from the nanoparticle. When combined with the increased radiolysis enhancement ratio at further distances from the nanoparticle this offered a promising pathway for enhancing biological effect at greater ranges. Despite the short range of direct damage this could potentially allow damage enhancement to DNA within the nucleus from GNPs in the cytoplasm. It was also found that the greatest enhancement around the nanoparticle occurred towards the proton source due to greater scattering of electrons within the GNP than the WNP. Simulating the interactions of the incident protons within water before reaching the GNP resulted in a slight reduction in enhancement due to the absorption of incident electrons produced by proton interactions within the water. A slight reduction in yield for short distances in the forward direction was due to shielding from the incident electrons caused by the GNP. Having multiple nanoparticles in proximity results in a large local dose and reactive species yield as well as extending the reactive species yield envelopes between the nanoparticles due to the overlap of the radial falloff from the nanoparticles. However, simulations showed that a significant reduction in yield of up to 17% for two nanoparticles and 60% for a nanoparticle cluster occurs due to the increased absorption from nanoparticles in proximity. The benefit of the increased total dose to water and resulting radiolysis yields for diffuse GNPs must be balanced against the potential enhanced local effect for clusters if they can be positioned close to a biological target. For GNP enhanced proton therapy it will be necessary to consider the size of the nanoparticles, how they cluster within the tumor cells and the incident proton energies to ensure that the desired radiosensitizing effect is delivered and that potential radioprotective effects from increased absorption and shadowing of the proton beam are minimized within tumor cells.

## Figures and Tables

**Table 1:** Incident proton beam data. D_PB_, D_BO_ and D_SEP_ are defined in Figure 12.

Simulation		D_PB_ (nm)	D_BO_ (nm)	Energy (MeV)	Number of Protons (GNP)	Number of Protons (WNP)
**Single**	Narrow beam	15	7.5	5	1.5 × 10^5^	6 × 10^5^
	Wide beam	400	500	5	3 × 10^7^	3.5 × 10^7^
	Cluster comparison	1000	1000	5	1.3 × 10^8^	-
Proximity	Perpendicular (D_SEP_ = 0.5–100 nm)	400	500	5	1.6 × 10^7^	3 × 10^7^
	Perpendicular (D_SEP_ = 200, 350 nm)	1000	500	5	7.5 × 10^7^	1.4 × 10^8^
	Parallel (D_SEP_ = 0.5–500 nm)	400	500	5	1.6 × 10^7^	3 × 10^7^
Cluster	-	1000	1000	5	2.3 × 10^5^	2.8 × 10^5^

**Table 2:** Formulas for relative dose and yield calculations. D_SEP_ is the separation distance between nanoparticles, V_O_ is the dose/yield for a GNP at the origin and V_D_ is the dose/yield for a single GNP at the position where the nanoparticle displaced in the positive direction along the separation axis would be for a separation distance of D_SEP_. The layout of the GNPs is shown in Figure 12.

Simulation		Weighted Yield for Absorption Calculation
**Proximity**	Perpendicular (D_SEP_ = 0.5–100 nm)	$2V_{O}+\left(D_{SEP}/100\right)\left(2\left(V_{100}-V_{O}\right)\right)$
	Perpendicular (D_SEP_ = 200, 350 nm)	$2V_{D}$
	Parallel (D_SEP_ = 0.5–500 nm)	$2V_{O}+\left(D_{SEP}/500\right)\left(V_{500}-V_{O}\right)$

## Abbreviations

GNP	Gold nanoparticle
WNP	Water nanoparticle
LET	Linear energy transfer
RBE	Relative biological effectiveness
LEM	Local effect model
DEF	Dose enhancement factor
REF	Radiolysis enhancement factor
EPR	Enhanced permeation and retention
Z	Atomic number
